# Toughening Polylactide Stereocomplex by Injection Molding with Thermoplastic Starch and Chain Extender

**DOI:** 10.3390/polym15092055

**Published:** 2023-04-26

**Authors:** Yottha Srithep, Dutchanee Pholharn, Patnarin Worajittiphon, Keartisak Sriprateep, Onpreeya Veang-in, John Morris

**Affiliations:** 1 Manufacturing and Materials Research Unit, Department of Manufacturing Engineering, Faculty of Engineering, Mahasarakham University, Mahasarakham 44150, Thailand; keartisaks@hotmail.com (K.S.); onpreeya.vea@msu.ac.th (O.V.-i.); 2 Department of Rubber and Polymer Technology, Faculty of Science and Technology, Rajabhat Mahasarakham University, Mahasarakham 44000, Thailand; dutchanee.ph@rmu.ac.th; 3 Department of Chemistry, Faculty of Science, Chiang Mai University, Chiang Mai 50200, Thailand; patnarin156@yahoo.com; 4 Center of Excellence in Materials Science and Technology, Chiang Mai University, Chiang Mai 50200, Thailand; 5 School of Industrial Education and Technology, King Mongkut’s Institute of Technology Ladkrabang, Bangkok 10520, Thailand; john.mo@kmitl.ac.th

**Keywords:** polylactide stereocomplex, thermoplastic starch, chain extender

## Abstract

The high cost, low heat resistance, and brittleness of poly(L-lactide) (PLLA) is a significant drawback that inhibits its diffusion into many industrial applications. These weaknesses were solved by forming a polylactide stereocomplex (ST) and blending it with thermoplastic starch (TPS). We blended poly (L-lactide)(PLLA), up to 30% thermoplastic starch, and a chain extender (2%) in an internal mixer, which was then hand-mixed with poly (D-lactide)(PDLA) and injection molded to form specimens, in order to study mechanical, thermal, and crystallization behavior. Differential scanning calorimetry (DSC) and wide-angle X-ray diffraction (XRD) demonstrated that the stereocomplex structures were still formed despite the added TPS and showed melting points ~55 °C higher than neat PLLA. Furthermore, stereocomplex crystallinity decreased with the increased TPS content. Dynamic mechanical analysis revealed that ST improved PLLA heat resistance, and tensile testing suggested that the TPS improved the elongation-at-break of ST. Moreover, the chain extender reduced the degradation of ST/TPS blends and generally improved ST/TPS composites’ mechanical properties.

## 1. Introduction

Polylactide (PLA) demonstrates good mechanical, optical, and barrier properties and degrades naturally [1]: it has two isomers, poly(L-lactide) (PLLA) and poly(D-lactide) (PDLA). Blending the PLLA and PDLA enhanced thermal properties and the resistance to hydrolysis, forming a polylactide stereocomplex (ST), which had a 220 °C melting temperature, ~50 °C higher than either isomer [2,3]. For a variety of melt processing and applications, additives that precisely alter the stereocomplex properties of polylactide are still necessary. In addition, optical purity, polymer chain length, and structure (e.g., degree of branching) affect the amount of stereocomplex formed [4]. However, compared to commodity polymers, PLA has a relatively high production cost and is brittle: developments in PLA composites to overcome these properties have been reviewed by Li et al. [5] and Zaaba and Ismail [6].

Increasing PLA toughness and ductility has been addressed by multiple techniques, including plasticization, copolymerization, and melt blending with various tough polymers [7]. Plasticization is a cost-effective process, but plasticizer migration must be considered. Physically mixing PLA with ductile and flexible polymers remains an intriguing option [8]. Improving PLA’s toughness and ductility by blending it with other polymers, including polyethylene [9], polyethylene-octene copolymer [10], and synthetic rubbers, for example nitrile butadiene rubber and ethylene propylene rubber, has been investigated [11].

Blending starch into PLA reduced material costs and increased degradation rates [12]. However, PLA with added starch composites became more brittle due to the coarse structure and reduced interfacial adhesion [13]. Moreover, since PLA is hydrophobic and starch is hydrophilic, the two substances interact in quite distinct ways [14]. Plasticizing and gelatinizing starch before mixing with PLA has improved material adhesion [13]. This gelatinized starch or thermoplastic starch (TPS) is deformable and able to be dispersed under a flow, leading to a dispersed phase containing particles finer than the basic starch. Combining PLA and TPS can increase the flexibility and elongation at break, which can improve the toughness significantly. This is a practical and affordable approach [8,13]. However, TPS accelerated the thermal degradation of PLA due to hydrolysis. Further, PLA and TPS are incompatible—there is little interfacial adhesion because PLA is hydrophobic, whereas TPS is hydrophilic [15]. In recent years, numerous studies introduced different materials to enhance the properties of TPS and PLLA blends. For example, Acioli-Moura et al. [16] used methylenediphenyl diisocyanate, Xiong et al. [17] studied the use of hexamethylene diisocyanate (HDI), and Li et al. [12] used chain extenders to improve the properties of PLLA and TPS blends.

PLA melt strength properties have been improved by reactive blending with a chain extender, a styrene-acrylic multifunctional oligomeric agent known as Joncryl^®^ to form long chain branching PLA structures [18]. Joncryl^®^ has been commonly used as a chain extender in the recycling of polycondensation thermoplastics via a melt processing chain extension reaction [19,20]. Lendvai and Brenn [21] compared it with maleic anhydride and blocked hexamethylend diisocyanate (bHDI) and confirmed that it was the most effective of the three. A chain extender can restore the polylactide molecular weight during the melt processing [22,23]. In addition, Zhang et al. discovered that the addition of Joncryl^®^ greatly enhanced the film tensile strength, yield strength, and especially the elongation, with a 250 percent elongation of 70/30 (TPS/PLA) film [24].

Biodegradable polymers must naturally degrade in the environment: PLA and its blends degrade through multiple mechanisms, including hydrolysis, effect of light, microbes, and enzymes: the PLA degradation was reviewed by Zaaba and Jaafar [6].

However, there is no study blending polylactide stereocomplex with TPS and using a chain extender to enhance blend properties. Therefore, we hypothesized that

(a) The polylactide stereocomplex (ST) would have better thermal stability than PLLA;
(b) Thermoplastic starch would create a tougher polylactide stereocomplex;
(c) The multifunctional epoxide group of a chain extender would reduce the stereocomplex degradation and enhance the properties of ST/TPS blends.

PLLA, PDLA, TPS, and a chain extender were melt-blended and injection molded. Differential scanning calorimetry and XRD measured the fraction of the stereocomplex formed in the blends. Heat resistant and mechanical properties were used to evaluate the effects of adding the thermoplastic starch and a chain extender. Morphologies, revealed by SEM images, confirmed the cause of the observed improvements.

## 2. Materials and Methods

### 2.1. Materials

The PLLA L175 (M_w_ = 210 kg/mol, M_w_/M_n_ = 1.84, and GPC analysis based on polystyrene standard) and PDLA D070 (M_w_ = 73 kg/mol, M_w_/M_n_ = 1.52, and GPC analysis based on the polystyrene standard) were purchased from Total Corbion PLA (Thailand) Ltd., Rayong, Thailand. The natural rice starch was obtained from the Thai Flour Industry Co., Ltd., Bangkok, Thailand. Glycerin (99.9% pure) as a TPS plasticizer was purchased from Green Global Chemicals Public Company Limited, Bangkok, Thailand. The chain extender (BASF (Thai) Limited, Bangkok, Thailand, Joncryl^®^ ADR-4370) was an epoxy-functional styrene acrylic copolymer or oligomeric coupling agent.

### 2.2. Sample Preparation

To prepare TPS, natural rice starch, with 25% wt% glycerin, was mixed by hand and allowed to stand (25 ± 2 °C, 24 h). The mixture was then fed to a mixer (HAAKE Polylab OS system, Thermo Fisher Scientific, Waltham, MA, USA) and melt blended (60 rpm, 200 °C, 4 min). In this first stage, TPS was obtained.

PLLA and PDLA, in equal portions, were mixed with TPS (added at 15% and 30% wt%) and the chain extender (2% wt%) by melt blending and injection molding. Blends with stereocomplexes demonstrated significantly changed properties (already observed with additional TPS [25]). Differential scanning calorimetry and XRD analyses measured stereocomplex formation in the blends. In addition, static and dynamic mechanical properties were measured, and SEM images were used to confirm morphological changes.

In a second blending, PLLA pellets were dried (vacuum oven, 80 °C, five h) and then mixed with TPS (15 or 30 wt%). Additionally, a chain extender (2 wt%) was added to reduce the degradation of the stereocomplex in the blends—see Table 1—and blended in an internal mixer (HAAKE Polylab OS system, Thermo Fisher Scientific, Waltham, MA, USA, 60 rpm, 200 °C, 4 min). After discharge, the molten mixture was granulated.

PLLA or PLLA/TPS/CE were hand-mixed with PDLA following the compositions shown in Table 1. Blends were then injection molded (ING-58T, Chareon Tut Co., Ltd., Samutprakarn, Thailand) to form tensile testing bars (ASTM D638 Type I) and flexural bars (ASTM D790, 125 mm × 12.7 mm × 3.2 mm). The nozzle temperature of the injection molding machine was set at ~225 °C. Pure PLLA and TPS bars were prepared as references.

### 2.3. Material Characterization

#### 2.3.1. Gel Permeation Chromatography

The weight-average molecular weights (M_w_) for pure PLLA and PDLA were measured by gel permeation chromatography. A total of ~7.5 mg samples were dissolved in 3 mL of THF at room temperature and passed through a 0.2 mm PTFE membrane filter. Then, 100 mL specimens were injected into a Shimadzu RDI-10A chromatograph (Shimadzu Corporation, Tokyo, Japan) with a reflective index (RI) detector, with 1.0 mL/min flow, and calibrated with a polystyrene standard.

#### 2.3.2. Differential Scanning Calorimetry (DSC)

Thermal properties were determined by differential scanning calorimetry (DSC 4000, Perkin Elmer, Waltham, MA, USA). A total of 4–5 mg specimens in Al pans were heated from 0 to 250 °C at 10 °C/min. Temperatures for glass transition, T_g_, cold crystallization, T_cc_, homo-melting, T_m,hc_, and stereocomplex melting, T_m,st_, and associated enthalpies, ΔH_cc_, ΔH_m,hc_, and ΔH_m,st_, were recorded. The degree of crystallinity, X_c_, for both the homo- and stereocomplex-crystals was calculated from [26,27]:

(1) $$X_{c}\;(\%)=\frac{\Delta H_{m,\mathrm{hc}}+\Delta H_{m,\mathrm{st}}-\Delta H_{\mathrm{cc}}}{w\;\times \Delta H_{m\left(\mathrm{blend}\right)}^{0}}\;\times \;100\%$$

where the melting enthalpies, ΔH_m,hc_ and ΔH_m,st_, were measured for pure crystallites and stereocomplex crystallites, ΔH_cc_ is the cold crystallization enthalpy, and w is the mass fraction of the stereocomplex in the polymer blends. $H_{m\left(\mathrm{blend}\right)}^{0}$ is the theoretical melting enthalpy for perfect crystals, calculated from:

(2) $$H_{m\left(\mathrm{blend}\right)}^{0}=H_{m,\mathrm{hc}}^{0}\;\times \;{ƒ}_{\mathrm{hc}}+H_{m,\mathrm{st}}^{0}\;\times \;{ƒ}_{\mathrm{st}}$$

where $H_{m,\mathrm{hc}}^{0}$ and $H_{m,\mathrm{st}}^{0}$ are the enthalpies of homocrystallites (93.6 J/g) and stereocomplex crystallites (142 J/g). ƒhc and ƒst are the fractions of homo- and stereocomplex crystallites:

(3) $${ƒ}_{\mathrm{hc}}=\frac{\Delta H_{m,\mathrm{hc}}}{\Delta H_{m,\mathrm{hc}}+\Delta H_{m,\mathrm{st}}}$$

(4) $${ƒ}_{\mathrm{st}}=\frac{\Delta H_{m,\mathrm{st}}}{\Delta H_{m,\mathrm{hc}}+\Delta H_{m,\mathrm{st}}}$$

The stereocomplex crystallinity, X_st_, was calculated from:

X_st_ (%) = X_c_ × ƒ_st_ (5)

#### 2.3.3. X-ray Diffraction Analysis

X-ray diffraction (XRD) measurements used a Bruker/D8 Advance (BrukerBioSpin AG, Waltham, MA, USA) to investigate the crystal structure of the blended samples. XRD samples were taken from injection-molded specimens and mounted on the XRD platform for the analysis. Scans covered 2θ from 5° to 40° in the refraction mode at 2°/min, using a computer-controlled wide-angle mode goniometer. X-rays were generated in a sealed tube Cu Kα source and passed through a thin Ni filter.

#### 2.3.4. Scanning Electron Microscopy (SEM)

Images of fractured surfaces were captured with a scanning electron microscope (HITACHI TM4000Plus, Hitachi, Ltd., Tokyo, Japan, 10 kV acceleration voltage). Tensile testing bars were frozen in liquid nitrogen, fractured, and sputter-coated with a ~20 nm Au layer.

#### 2.3.5. Thermogravimetric Analysis (TGA)

Thermal data was recorded isothermally at a constant temperature (320 °C, 60 min) or non-isothermally with heating at a constant 10 °C/min rate up to 600 °C in a TGA 4000 system (Perkin-Elmer, Waltham, MA, USA). A total of ~10 mg of each sample (conditioned at 25 °C, 50% relative humidity) was used. The mass loss was recorded and normalized versus the initial mass.

#### 2.3.6. Dynamic Mechanical Analysis (DMA)

Dynamic mechanical properties were measured with a TA Q800 DMA machine (TA Instruments, New Castle, DE, USA) in three-point bending mode. Injection-molded parts were cut into specimens (~17.6 mm × ~12.7 mm × ~3.2 mm), which were heated at 3 °C/min from 30 °C to 150 °C and mounted so that they were deflected by 0.01% of their length at 1 Hz.

#### 2.3.7. Heat Resistance Analysis

A qualitative test for heat resistance used straight flexural bars, first set in a frame, heated at 100 °C for 30 min to observe a specimen deformation under its own weight.

Moreover, a dynamic mechanical analyzer (TA Instruments DMA Q800, New Castle, DE, USA) operated with three-point bending clamps in the DMA controlled force mode under a 0.45 MPa load. The deflection was recorded with a 2 °C/min heating rate from 30 to 100 °C.

#### 2.3.8. Tensile Testing

Tensile testing followed ASTM D638-10 in an NRI-TS501 universal testing apparatus (Narin Instrument Co., Ltd., Bangkok, Thailand). Tensile testing on all specimens used an initial 0.5 N load and a constant 10 mm/min crosshead speed. Means from five replicates were measured.

#### 2.3.9. Impact Testing

Following ASTM D256, notched Izod impact testing used injection-molded samples. Rectangular specimens measuring roughly 63.5 mm × 12.7 mm × 3.2 mm were cut. Five samples from each sample group were examined, and the mean results were reported.

## 3. Results

### 3.1. Injection Molding of Blends

**Figure 1:**
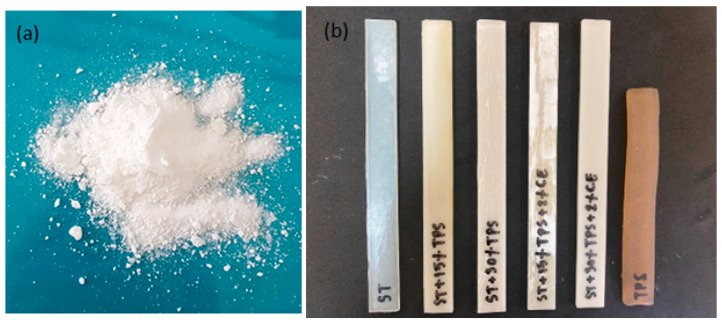
(**a**) Appearance of hand-mixed PLLA and PDLA, injection molded at 180 °C and (**b**) injection-molded samples formed at 225 °C with varying amounts of TPS and 2% chain extender.

When PLLA and PDLA were hand mixed and then injection molded at 180 °C, the materials stuck within the injection molding machine, and fine solid particles were extruded from the machine’s nozzle—see Figure 1a. This indicated that stereocomplex material, which had a melting point higher than 200 °C (cf. 3.2 and 3.3), was formed as the injection molding blended PLLA and PDLA. The particles had a high crystallization rate and solidified immediately at the 180 °C molding temperature [3]. However, when the molding temperature was 225 °C, tensile bars and flexural specimens could be produced. Figure 1b shows bars of as-molded blends. The TPS sample was brownish and had a high shrinkage rate, which could limit its utility [28]. The dark color was the result of slight thermal degradation. On the other hand, the blend of TPS with the stereocomplex did not shrink and was lighter in color. The color was observably lighter when the 2% chain extender (CE) was blended into the composites. This was attributed to the effect of the epoxy groups in the copolymer, which extended the chains, enhanced the molecular weight, and reduced the degradation of stereocomplex/TPS blends. Najafi et al. reported that the chain extender (Joncryl) significantly increased the PLA molecular weight [29].

### 3.2. Thermal Properties

**Figure 2:**
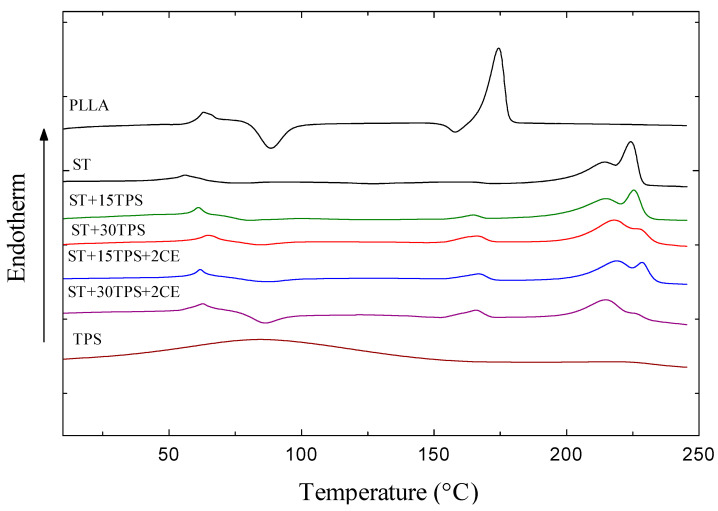
DSC melting curves for PLLA, ST, TPS, and ST/TPS/CE blends.

DSC measured crystallization and melting behaviors: the thermograms are shown in Figure 2, and extracted parameters are in Table 2. Figure 2 shows that PLLA exhibited three thermal steps: (1) glass transition, T_g_ ~60 °C, (2) cold crystallization (95–120 °C), and (3) endothermic fusion (melting peak, T_m_, maximum 155–175 °C). Cold crystallization was observed because, during injection molding, PLLA crystallization was hampered by the high cooling rate, so when PLLA was reheated during the DSC test, some mobility was recovered, and it crystallized again [30].

However, although the PLLA and PDLA blend (polylactide stereocomplex; ST) had a similar glass transition, T_g_ ~60 °C, endothermic peaks, observed from 208 to 230 °C, were assigned to the stereocomplex crystallite melting: since they appeared ~50 °C higher than the PLLA peak, they confirmed a complete stereocomplex crystallite formation, i.e., no homocrystallites formed. The T_m_ of pure ST was 224.1 °C, melting enthalpy 71.6 J/g, and degree of crystallinity, X_st_ = 50.4%—see Table 2. However, the cold crystallization transition of the stereocomplex almost disappeared, suggesting a higher crystallization rate for the stereocomplex than the pure PLLA after injection molding [3].

Figure 2 also shows thermograms of injection-molded polylactide stereocomplex (ST) blended with 15% and 30% TPS. The samples show two T_m_ peaks at 164–165 °C, assigned to melting homocrystallites, ΔT_m,hc_, and the melting of stereocomplex crystallites, ΔT_m,sc_, at 214–228 °C. The homocrystallite melting enthalpies were much lower than stereocomplex crystallite melting enthalpies, indicating the forming of mostly stereocomplex crystals. Since the TPS was mostly amorphous, the ST-TPS blends decreased the stereocomplex melting enthalpy. Therefore, the higher TPS content led to a lower degree of crystallinity.

Moreover, the effect of 2% CE on the thermal properties of ST/TPS blends is also shown in Figure 2 and Table 2. After introducing the chain extender, the increased molecular weight of the stereocomplex restricted chain mobility. The lower crystalline content was expected, as some of the PLA chains appeared in grafted structures, with multiple chains attached to a single chain extender molecule. For ST + 30TPS blends without a chain extender, the crystallinity of stereocomplex crystallites, X_st_, was estimated at 45%, but adding the chain extender decreased X_st_ to ~30%.

### 3.3. XRD Analysis

**Figure 3:**
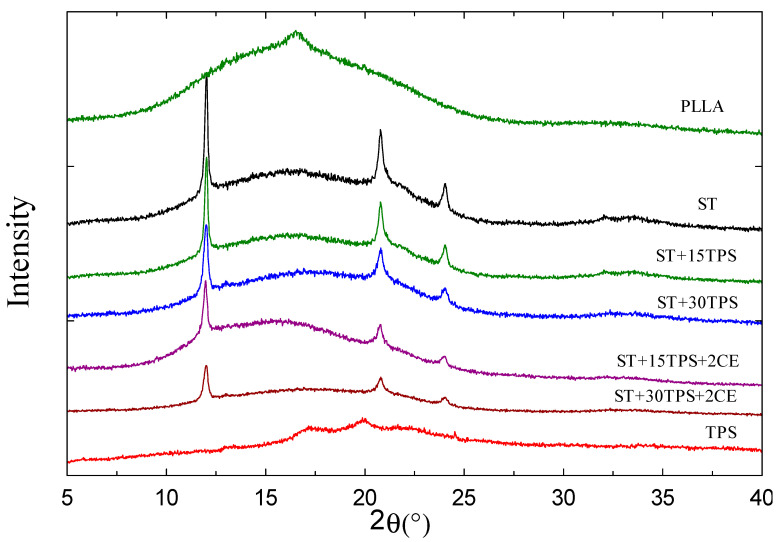
XRD profiles of PLA and ST/starch blends without CE and with 2% CE content.

The crystal structure was determined from XRD spectra at room temperature. Figure 3 shows that pure PLLA was essentially amorphous: a broad halo was observed, 2θ ≈ 16°, with a small peak at ~16.2° indicating a small amount of crystalline PLA [30]. The high cooling rate during injection molding partially prevented PLLA from crystallizing.

However, the stereocomplexes showed three distinct peaks (2θ ~11.6°, ~20.6°, and ~23.5°) assigned to stereocomplex crystal planes [3]: these positions matched reported values [1,31]. This phenomenon also demonstrated that adding PDLA significantly increased their crystallization rate [32]. Stereocomplexes with added TPS showed the same peaks, but their intensity decreased with the increasing TPS content. According to Li et al. [33], the addition of TPS decreased the PLA melting enthalpy gradually. Further, when the chain extender was added, all peaks gradually became smaller. As the chain extender was added to ST/TPS composites, the increased PLA molecular weight also slowed crystallization and led to a lower final crystallinity. As multiple PLA chains were grafted to a single chain extender molecule, the crystalline content decreased as expected [12]. This confirmed a similar degree of crystallinity to that observed in DSC thermograms.

### 3.4. Thermal Stability

**Figure 4:**
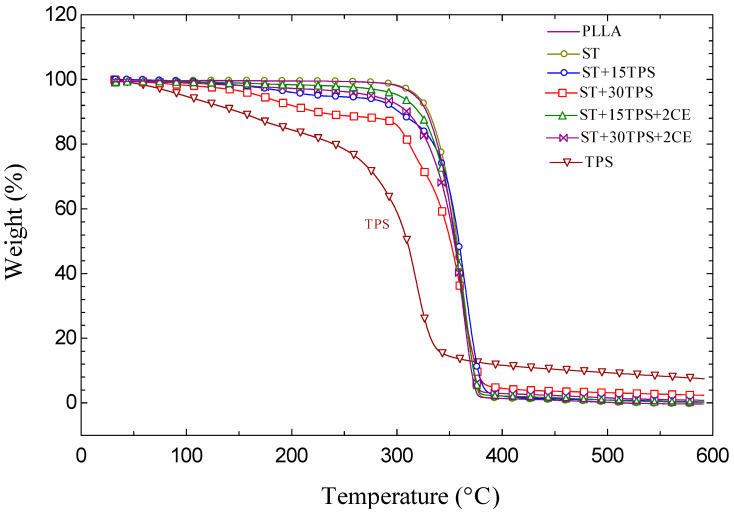
Nonisothermal measurement of the percentage of remaining weight of PLLA, TPS, ST/TPS/CE at a constant 10 °C/min heating rate.

PLA and TPS were very sensitive to high temperatures. Thermogravimetric analysis (TGA) curves were used to investigate thermal stability and decomposition. The remaining weight of the injection-molded samples measured non-isothermally is shown in Figure 4. TGA results confirmed that adding TPS lead to increased degradation. The onset degradation temperatures of the ST/TPS composites decreased with the addition of TPS. TPS had an approximately 10% char yield above 400 °C. We conducted the isothermal measurements at constant 325 °C holding temperatures to explore the thermal degradation behavior and stability in more detail.

**Figure 5:**
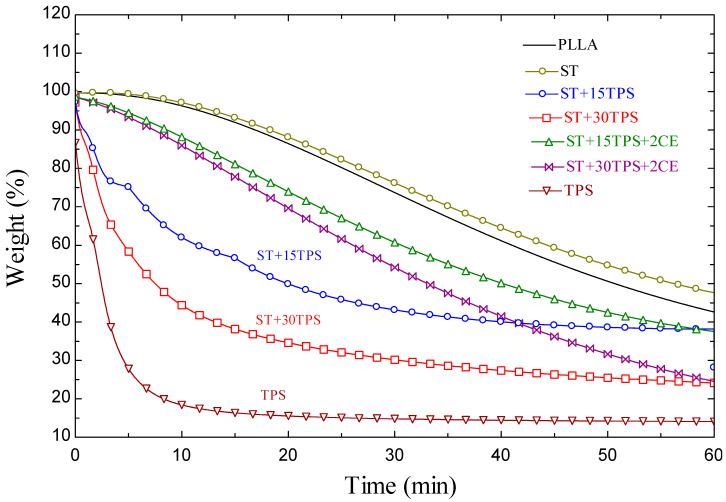
Fraction of the remaining mass of injection-molded samples measured isothermally at 320 °C.

Isothermal measurements used a constant holding temperature of 320 °C. Figure 5 shows the remaining fractional mass versus time. Table 3 lists the temperatures derived from the TGA thermograms corresponding to 30% mass loss. The blend of PLLA/PDLA (ST) had a slightly higher thermal stability and lower mass loss. Accordingly, the PLLA thermal degradation resistance was enhanced by the stereocomplex structure. The 30% (T_30%_) weight loss was reached at 32.9 min for PLLA, but it took 35.2 min for ST. The interaction between PLLA and PDLA chains may arise from their 10_3_ or 3_1_ helical conformations in the crystallized state. In these helical states, the interaction between the left and right-handed helices of PLLA and PDLA in their blended film must be stronger than that between chains with the same helical direction in pure PLLA and PDLA, resulting in decreased chain mobility and enhanced thermal stability of the stereocomplex film. Similar behavior in isothermal degradation tests of PLLA and the stereocomplex were reported previously [3,34].

Figure 5 also shows the TPS thermal stability. The TPS mass loss was notable, but it was expected, since naturally sourced components burn at lower temperatures than synthetic ones, such as PLA. For ST/TPS blends, the mass loss lay between that for ST and TPS and was more pronounced when the amount of TPS was higher. The T_30%_ of ST + 15%TPS was ~6.7 min, whereas it was ~2.6 min for ST + 30%TPS. Petinakis et al. found that small molecules, including CO, CO_2_, H_2_O, CH_4_, C_2_H_4_, and CH_2_O, were produced as starch decomposed, and they concluded that these molecules triggered the PLA chain scission [7]. Shi et al. reported that with the increased TPS content, the thermal decomposition of TPS also increased, whereas the decomposition temperature of PLA/TPS decreased [35].

When the 2% chain extender was added to the ST/TPS blend, thermal stability dramatically increased. The time at 30% mass loss, T_30%_, rose from 2.6 min for a sample without the chain extender (ST/30TPS) to 19.9 min with the chain extender (ST/30TPS/CE); thus, it reduced degradation in the ST/TPS blend.

### 3.5. Morphology

**Figure 6:**
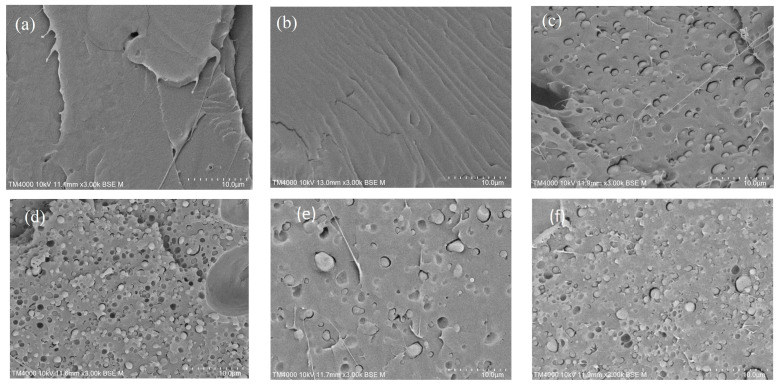
Micrographs: pure materials (**a**) stereocomplex—ST and (**b**) TPS; sterecomplexes plus TPS; (**c**) ST + 15%TPS; (**d**) ST + 30%TPS; plus chain extender (**e**) ST + 15%TPS + 2%CE; and (**f**) ST + 30%TPS + 2%CE.

Figure 6 shows stereocomplex blend (ST/TPS/CE) SEM images. For the pure stereocomplex and TPS, the observed smooth fracture surfaces in Figure 6a,b were typical of brittle fractures caused by freezing. In contrast, the stereocomplex plus TPS blends (ST + 15%TPS (Figure 6c) and ST + 30%TPS (Figure 6d) had distinct phases, confirming previous reports [12]. A coarse dispersion was observed with particle sizes ranging from 1–2 µm. We checked whether the epoxy-based chain extender interacted with the hydroxyl groups on the TPS macromolecules and thus played a role at the blend interface. Moreover, adding the 2% chain extender showed slightly increased compatibility, so the dispersed phase became slightly less extensive (see Figure 6e—ST + 15%TPS + 2%CE and 6f—ST + 30%TPS + 2%CE). Similar effects were observed with PLLA/TPS blended with a chain extender, which mostly reacted with PLLA chain ends and did not create any graft copolymer of PLLA with TPS [12].

### 3.6. Mechanical Properties

**Figure 7:**
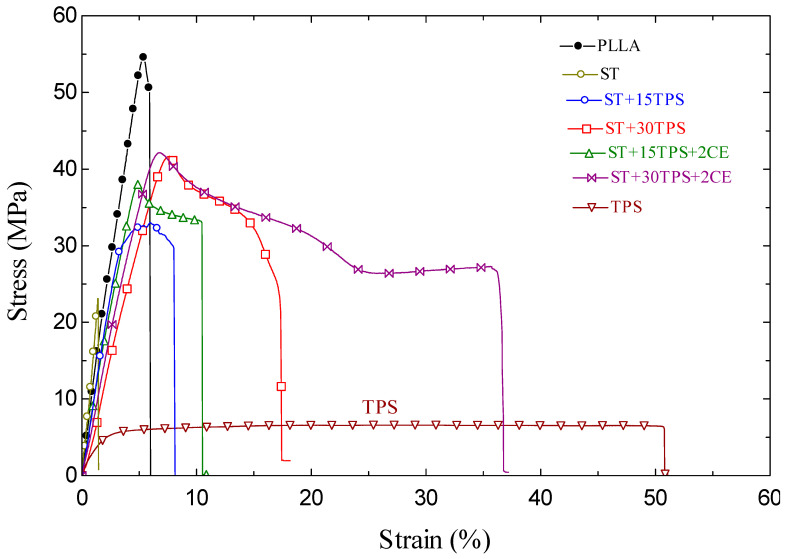
Stress vs. strain for stereocomplex and TPS blends: “+2CE” labels samples with 2wt% chain extender added.

Representative stress–strain curves of ST blended with the starch and chain extender are shown in Figure 7. Tensile modulus, tensile strength, and strain-at-break were measured—see Table 4. PLLA had a higher molecular weight than PDLA: the weight average M_w_ of PLLA was ~210 kg/mol, whereas it was lower for PDLA at ~73 kg/mol. Figure 6 and Table 4 show that PLLA had the highest tensile strength (54.9 MPa) and modulus. On the other hand, the tensile strength of injection-molded PLLA blended with the PDLA blend (stereocomplex) was 22.9 MPa. The reduction in tensile strength, and strain-at-break of PLLA, when blended with 50% PDLA, was attributed to the lower PDLA molecular weight. Moreover, Tsuji and Ikada [36] reported a significant difference in film shrinkage between the PLLA/PDLA blend (stereocomplex) and nonblended film. The blended film showed a diameter shrinkage of 15%, while the nonblended film shrank only 3%, which was attributed to the higher density of the microcrystallites in stereocomplex compared to spherulites in nonblended samples. In this study, this shrinkage in stereocomplex samples caused a warp in injection-molded samples and reduced the tensile properties compared to neat PLLA.

The stereocomplex tensile strength and strain-at-break increased when blending with TPS, i.e., with TPS in the stereocomplex, the films were tougher. Przybytek et al. [25] also noted that the thermoplastic starch embedded in the matrix increased flexibility and reduced strength. The increased strength of the stereocomplex when blending with TPS was attributed to the lower amount of PDLA, which had a lower molecular weight. Moreover, TPS reduced the shrinkage and warp due to stereocomplex crystallites. The tensile strength of the stereocomplex increased from 22.9 MPa to 41.2 MPa after blending with 30% TPS.

Furthermore, adding the chain extender improved the tensile properties of ST/TPS blends. In general, PLA with an added chain extender was found to have a higher molecular weight and better mechanical properties [22]: chain extenders react and rejoin the broken chains of both hydroxyl (-OH) and carboxyl groups (-COOH) of PLA during melt processing, leading to an improvement in tensile properties. For ST + 15%TPS, the ultimate tensile strength was 32.6 MPa, but with the chain extender, it increased to 38.2 MPa.

Table 4 also shows the impact strength of the seven ST blends and TPS. As observed, the mixture containing a greater proportion of TPS displayed a greater impact strength. Similar results were reported in the earlier study by Przybytek et al., who showed that mixing TPS and PLA led to a small increase in the impact strength [25]. Additionally, the blend of chain extenders (CE) and ST/TPS increased the impact strength. Zhang et al. discovered that the chain extender (Joncryl^®^) enhanced the mechanical characteristics of PLA/TPS blends [24]. The increased mechanical properties in ST/TPS blends with chain extenders (CE) were attributed to the decrease in ST degradation and improvement in the interfacial adhesion between ST and TPS.

### 3.7. DMA Analysis

**Figure 8:**
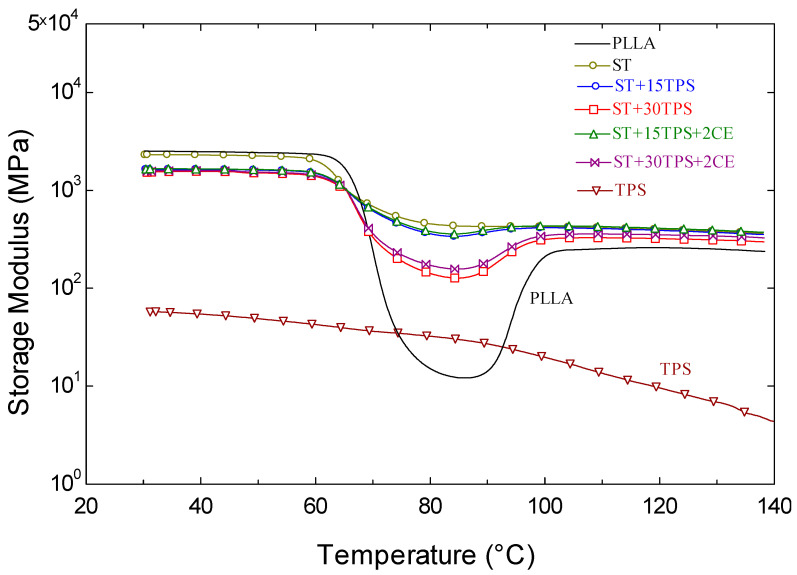
Storage moduli of polylactide stereocomplex (ST) blends vs. temperature.

Figure 8 displays storage moduli vs. temperature curves. With the increasing temperature, PLLA first exhibited a glassy state, then a glass transition and cold crystallization. In the glassy state, −30 to 60 °C, PLLA exhibited the highest storage modulus, which later decreased between 60 and 80 °C, in the glass transition, to a more flexible state [30]. Then, between 90 and 110 °C, the modulus started to increase due to the cold crystallization of the PLLA (cf. Figure 2): this increase in crystallinity increased the PLLA rigidity.

Whereas the stereocomplex had a slightly decreased storage modulus, matching the tensile properties. However, the subsequent drop in the storage modulus in PLLA disappeared with the stereocomplex formation. This suggested that PDLA allowed crystallization during injection molding. Srithep et al. reported that PDLA strongly affected the PLLA crystallization [32].

Figure 8 also shows the storage moduli of TPS and ST/TPS blends. TPS had the lowest storage modulus, did not show any phase transitions in the measured temperature range, and gradually decreased the modulus with temperature. For the ST/TPS blends, the storage modulus did not differ significantly at room temperature, but more TPS led to a decrease in the storage modulus during the glass transition region—60–80 °C, which indicated a higher cold crystallization enthalpy (cf. Table 2). Moreover, as shown in Figure 7, the addition of a chain extender led to similar trends to those without it, although it slightly increased the storage moduli of the ST/TPS blends.

**Figure 9:**
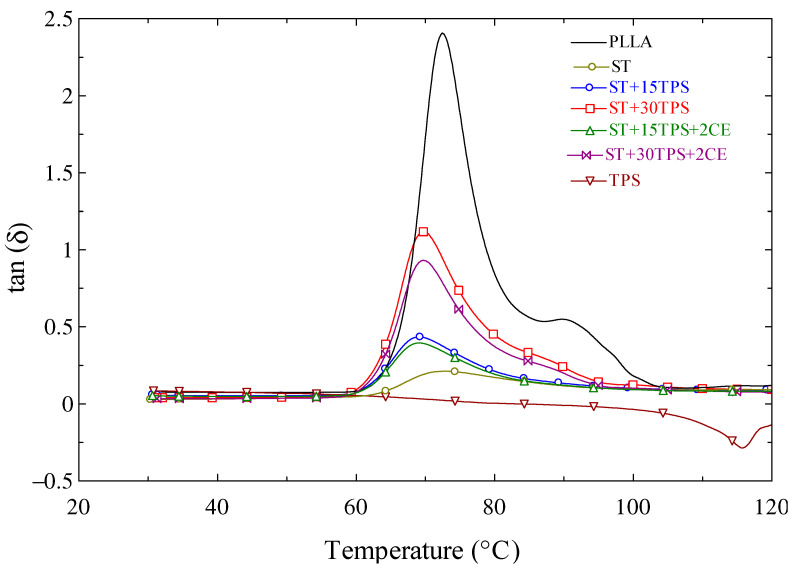
Tan δ curves of polylactide stereocomplex (ST) blends vs. temperature.

Figure 9 shows the tan δ curves. The area below the tan peak shows the materials’ damping capacity to absorb and disperse energy. As can be observed in Figure 9, the highly crystalline ST had less energy absorbing and damping ability than the amorphous specimens. The increased crystallinity increased the rigidity of the specimens. Additionally, the ST specimen area beneath the tan δ peak grew as the TPS content increased, indicating that TPS was less effective at absorbing energy than ST. In the ST/TPS composites, adding 2% chain extender generated a similar change in the region behind the tan δ peak. The peak of the tan δ curves in Figure 9 also indicates the glass transition temperature of the blends. One can also observe that the glass transition temperature from the DMA experiment of the blended samples was similar, around 72 °C, which was higher than that from the DSC experiment because the DSC heating rate was faster, at 10 °C/min.

### 3.8. Heat Resistance

**Figure 10:**
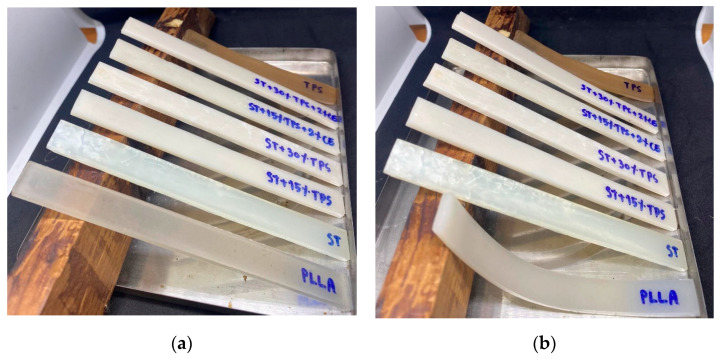
Heat resistance of injection-molded PLLA, TPS, and ST/TPS blends (**a**) as-fabricated; (**b**) after heating at 100 °C for 30 min.

Injection-molded samples were set up as shown in Figure 10a and placed in an oven at 80 °C to observe the heat resistance and deformation. Figure 10b shows that only pure PLLA (the first specimen), which had the lowest degree of crystallinity, obviously deformed at 80 °C. However, no stereocomplex or blend with TPS deformed in this test. This indicates that the combination of PLLA and PDLA led to better heat resistance, as the stereocomplex formation caused a higher degree of crystallinity. Other ST/TPS/CE samples also showed very little or no deformation, suggesting a better heat-resistant behavior.

**Figure 11:**
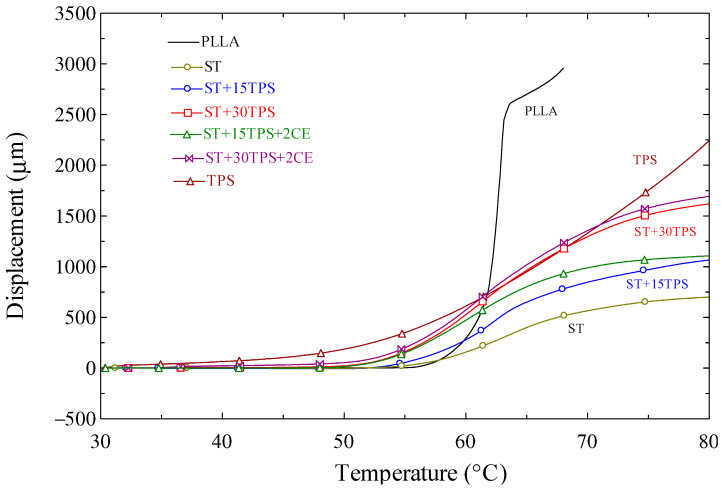
Deflection of the PLLA, TPS, ST blended with TPS, and chain extender under a 0.45 MPa load as the temperature increased.

Moreover, Figure 11 depicts the deflection of the PLLA, TPS, ST blended with TPS, and chain extender under a 0.45 MPa load as the temperature increased. It is evident that the PLLA deflected rapidly at around 60 °C, which corresponds to the glass transition temperature of PLLA (c.f. Table 2). This result is reasonable because the deflection temperature of a polymer with low crystallinity is close to its T_g_ [37]. On the other hand, TPS deflected progressively as the temperature increased. From Figure 8, at 80 °C, the TPS modulus was higher than PLLA. Therefore, as Figure 10 shows, TPS did not clearly deform like PLLA at 80 °C. For the blend of ST and TPS, the higher amount of TPS demonstrated a higher deflection but the addition of chain extender did not cause a significant difference in the heat resistance.

## 4. Conclusions

Samples made from the polylactide stereocomplex blended with the thermoplastic starch (TPS) and chain extender were prepared by injection molding. Despite adding up to 30% TPS, the PLA stereo composites formed a stable stereo composite structure, and the melting point was 55 °C higher than that of pure PLLA. Wide angle X-ray diffraction demonstrated that the crystallinity of the stereocomplex decreased with increasing TPS content and further decreased when a chain extender was added. With the increased TPS content, the tensile stress increased, and the strain-at-break increased. With the addition of the 2% chain extender in the ST + 30%TPS sample, the elongation at the break increased steadily, reaching 36%. The thermal stability of the stereocomplex and TPS blends was improved through chain elongation reactions, thus improving the mechanical properties of the composites.

## Figures and Tables

**Table 1:** Compositions of blended samples.

Sample	PLLA (wt%)	PDLA (wt%)	TPS (wt%)
PLLA	100	0	0
TPS	0	0	100
ST	50	50	0
ST + 15%TPS	42.5	42.5	15
ST + 30%TPS	35	35	30
ST + 15%TPS + 2%CE	41.5	41.5	15
ST + 30%TPS + 2%CE	34	34	30

**Table 2:** Thermal characteristics of the blends.

Sample	T_g_ (°C)	Cold Crystallization		Melting Homocrystal (hc)		Melting Stereocomplex Crystal (st)		%X_c_ ^a^	%X_st_ ^b^
		T_cc_ (°C)	∆H_cc_ (J/g)	T_m,hc_ (°C)	∆H_m,hc_ (J/g)	T_m,st_ (°C)	∆H_m,st_ (J/g)		
PLLA	61.8	87.4	22.2	173.4	49.5	-	-	29.0	-
ST	56.3	74.9	2.6	-	-	224.1	71.6	48.2	48.2
ST + 15TPS	58.5	79.9	7.2	164.9	3.0	225.4	61.6	48.3	46.1
ST + 30TPS	61.4	84.5	7.0	165.8	7.3	228.0	48.9	51.8	45.1
ST + 15TPS + 2CE	59.1	85.8	9.7	167.1	6.0	228.9	51.2	40.8	36.5
ST + 30TPS + 2CE	58.0	86.0	16.9	165.8	7.4	214.9	42.9	35.4	30.2
TPS	-	-	-	84.9	158.2	-	-		

^a^ calculated from Equation (1). ^b^ calculated from Equation (5).

**Table 3:** Thermal loss points for ST, TPS, and ST/TPS blends.

Sample	T_30%_ (min)
PLLA	32.9
ST	35.2
ST + 15TPS	6.7
ST + 30TPS	2.6
ST + 15TPS + 2CE	22.7
ST + 30TPS + 2CE	19.9
TPS	0.7

**Table 4:** Mechanical properties of ST/TPS blends.

Sample	Ultimate Tensile Strength (MPa)	Tensile Modulus (MPa)	Strain at Break (%)	Impact Strength (kJ/m^2^)
PLLA	54.9 ± 3.2	2734 ± 217	6.0 ± 0.3	1.4 ± 0.08
ST	22.9 ± 1.7	2866 ± 234	1.5 ± 0.2	0.9 ± 0.05
ST + 15TPS	32.6 ± 2.6	1993 ± 147	8.2 ± 0.5	2.1 ± 0.1
ST + 30TPS	41.2 ± 3.4	1751 ± 132	17.5 ± 2.4	4.2 ± 0.2
ST + 15TPS + 2CE	38.2 ± 2.5	22,340 ± 202	10.5 ± 0.8	3.2 ± 0.2
ST + 30TPS + 2CE	41.7 ± 2.5	1939 ± 157	36.7 ± 2.7	8.1 ± 0.4
TPS	6.7 ± 0.3	738 ± 54	50.8 ± 4.2	NB ^1^

^1^ NB, not broken.

## Data Availability

All data included in this study are available upon request by contacting the corresponding author.
